# Mechanistic insights into *NFIX* frameshift mutations in Malan syndrome: proteasomal degradation-mediated haploinsufficiency

**DOI:** 10.3389/fgene.2025.1648420

**Published:** 2025-11-07

**Authors:** Yan Zhou, Linbing Zou, Yaoyao Li

**Affiliations:** 1 Department of Basic Medicine, School of Medicine, Jingchu University of Technology, Jingmen, China; 2 Hefei Maternal and Child Health Hospital Reproductive Medicine Center, Hefei, China

**Keywords:** malan syndrome, intellectual disability, NFIX, ubiquitin-proteasome pathway, haploinsufficiency

## Abstract

**Objective:**

To investigated the pathogenic mechanism of *NFIX* frameshift mutations in Malan syndrome.

**Methods:**

Reviewed the clinical diagnosis and treatment processes of the Malan syndrome proband, analyzing the relationship between *NFIX* frameshift mutation genotypes and clinical phenotypes, and the inheritance pattern. To analyzed the functional domain where the mutation was located and the conservation of the mutated amino acid residue, thereby elucidating the potential impact of the mutation on the protein. Validated effects on pre-mRNA splicing using RDDC^SC^, SpliceAI, and FF databases. Assessed variant pathogenicity via MutationTaster, PolyPhen-2, and VarCards. Constructed wild-type/mutant plasmids, transfected to HEK293T cells, and quantified *NFIX* mRNA and protein expression levels via qPCR and Western blot. Analyzed degradation pathways using ubiquitin-proteasome inhibitor MG132 and autophagy-lysosome inhibitor Chloroquine (CQ).

**Results:**

The proband exhibited intellectual disability, distinctive facial features, ocular abnormalities, scoliosis, and primary infertility. A *de novo* mutation in *NFIX* (c.164delC, p.Ala55Glyfs*2) associated with these phenotypes was identified. Neither the proband’s father nor his mother was found to have this mutation. Parental testing confirmed *de novo* inheritance. The amino acid at position 55 was highly conserved and had been Alanine in 5 species. Results from databases including RDDC^SC^, SpliceAI, and FF indicated that the *NFIX* c.164delC p.Ala55Glyfs*2 mutation did not affect splicing function. Predictions by MutationTaster and PolyPhen-2 classified the c.707G>A p.Arg236Gln mutation as “damaging,” suggesting an altered amino acid sequence, frameshift mutation, NMD, and potential modification of protein characteristics. Quantitative real-time PCR (qPCR) analysis detected comparable mRNA levels between mutant and wild-type strains. In contrast, Western blotting revealed significantly diminished protein expression in the mutant (*P* < 0.05), suggesting post-transcriptional regulation effects. Results from protein degradation pathway analysis demonstrated that the truncated protein generated after mutation was degraded via the ubiquitin-proteasome pathway.

**Conclusion:**

The *NFIX* c.164delC p.Ala55Glyfs*2 frameshift mutation did not significantly affect mRNA expression levels, but induced protein degradation via the ubiquitin-proteasome pathway, resulting in haploinsufficiency and ultimately causing Malan syndrome.

## 1 Introduction

Malan syndrome is an autosomal dominant disorder characterized by overgrowth, neurodevelopmental disorders, and connective tissue abnormalities, first reported by Malan in 2010 in three unrelated patients (Malan et al., 2010). These patients exhibited clinical manifestations including postnatal overgrowth, macrocephaly, advanced bone age, a long and narrow face with a high forehead, marfanoid habitus, scoliosis, behavioral abnormalities such as anxiety, and intellectual disability. The characteristic clinical phenotypes comprise four components: (1) Growth abnormalities: Postnatal overgrowth resembling Sotos syndrome, presenting as significantly taller stature than peers and abnormally slender limbs (Klaassens et al., 2015); (2) Craniofacial abnormalities. Manifestations include: macrocephaly, frontal bossing, high anterior hairline, and elongated facial shape, with prognathism observed in some cases (Zenker et al., 2019); (3) Neurocognitive and developmental impairments: Manifested by mild-to-moderate intellectual disability, compromised learning abilities and cognitive function, with gross motor and fine motor skill impairments during infancy and early childhood (Uzman et al., 2023); (4) Complications. Spinal scoliosis and joint hyperlaxity may occur in a subset of patients (Priolo et al., 1993). These clinical phenotypes demonstrated considerable diversity and complexity, with growth deformities being the most prevalent presentation.

It has been established that mutation of *NFIX* (Nuclear Factor I X; OMIM 164005) at chromosomal locus 19p13 underlies the pathogenesis of Malan syndrome (OMIM 614753) (Priolo et al., 1993). *NFIX* belongs to a family of CCAAT-binding transcription factors (Santoro et al., 1988) that initiate transcription of various genes, including vertebrate and viral genes. The nuclear factor I protein encoded by *NFIX* is a ubiquitous 47-kDa dimeric DNA-binding protein recognizing the sequence TGG(C/A)N(5)GCCAA, which exists in multiple DNA viruses and the human genome (Singh et al., 2009). *In vitro*, NFIX proteins can stimulate the initiation of adenoviral DNA replication and synergize with other factors (such as estrogen receptor ESR) to stimulate gene transcription (Malaymar et al., 2025; Chen et al., 2020). *NFIX* is expressed across various human tissues and cells, with more prominent expression in the central and peripheral nervous systems (O'Connor et al., 2015; Fraser et al., 2017). The *NFIX* gene plays critical roles in the development of the cerebral cortex, cerebellum, and spinal cord, influencing neuronal proliferation, differentiation, and migration (Campbell et al., 2008); participates in skeletal muscle formation and bone development (Qiu et al., 2024); and is also associated with tumorigenesis (Ribeiro et al., 2023). Cumulative evidence has revealed a spectrum of pathogenic NFIX variants underlying Malan syndrome, encompassing: frameshift (Osh et al., 2017), deletion (Bellucco et al., 2019), nonsense, and splice-site mutations (Yoneda et al., 2012). These mutations manifest clinically with Sotos-like phenotypes (intellectual disability and macrocephaly) (Martinez et al., 2015), and may also be accompanied by additional features including aberrant behaviors, ophthalmic abnormalities, and gastrointestinal disturbances. Given the limited number of cases, it was impossible to establish whether genotype-phenotype correlations existed.

Although the pathogenic mechanisms of Malan syndrome have not been fully elucidated, most researchers contend that haploinsufficiency and point mutations in the *NFIX* gene constitute the major cause of this disorder; however, specific mechanisms require further investigation. In this study, the proband exhibited clinical features including intellectual disability, dysmorphic facial features, ocular anomalies, scoliosis and primary infertility. A heterozygous *NFIX* mutation associated with these phenotypes was identified, and parental testing confirmed its *de novo* origin. Since the proband’s husband also had primary infertility, the pathogenicity of the *NFIX* gene heterozygous mutation was evaluated to reduce the risk of offspring inheriting the genetic disease. This study expands the mutational spectrum of Malan syndrome while strengthening evidence for genotype-phenotype correlations. Furthermore, through *in vitro* expression assays and degradation pathway analyses, it was demonstrated that heterozygous *NFIX* mutations trigger proteasomal degradation, resulting in *NFIX* haploinsufficiency-ultimately driving the molecular pathogenesis of Malan syndrome. These findings provided a theoretical framework for clinical research on disease mechanisms.

## 2 Materials and methods

### 2.1 Clinical case

The proband presented to the Reproductive Medicine Center of Anhui Provincial Maternity and Child Health Care Hospital in 2023 due to primary infertility. Following admission, laboratory investigations were completed, including basic endocrine tests, hysterosalpingography, electrocardiogram (ECG), echocardiogram, and vaginal secretion analysis. The study obtained informed consent from the patient and was approved by the hospital’s Ethics Committee.

### 2.2 Whole exome sequencing

Peripheral blood (2 mL) was collected from the proband. Genomic DNA was extracted using a peripheral blood genomic DNA extraction kit, and its concentration was measured. The DNA was then sent to Shenzhen Huada Medical Laboratory for whole exome sequencing. First, the DNA was fragmented and a library was prepared. Next, the DNA was captured and enriched for target gene exons and adjacent splicing regions using the Roche KAPA HyperExome chip. Finally, variant detection was performed using the MGISEQ-2000 sequencing platform. Quality control metrics for sequencing data included: an average sequencing depth of the target region ≥180×, with >95% of sites achieving an average depth >20×. Sequencing fragments were aligned to the UCSC hg19 human reference genome using BWA, and duplicates were removed. GATK was employed for base quality score recalibration, single nucleotide variant (SNV) and insertion/deletion (INDEL) detection, and genotype calling. ExomeDepth was utilized for exon-level copy number variation detection. Variant pathogenicity classification followed the guidelines of the American College of Medical Genetics and Genomics (ACMG) and the Association for Molecular Pathology (AMP) for sequence variant interpretation, with reference to refinements from the ClinGen Sequence Variant Interpretation Working Group and the Association for Clinical Genomic Science (ACGS).

### 2.3 Mutation pathogenicity prediction

The functional domain of the mutation was analyzed by consulting NCBI and relevant literature. The PolyPhen-2 online tool (http://genetics.bwh.harvard.edu/pph2/) was utilized to assess the conservation of amino acids at the mutation site and elucidate potential impacts on protein function. Databases including RDDCSC (https://rddc.tsinghua-gd.org/search-middle?to=SplitToolModel), SpliceAI (https://spliceailookup.broadinstitute.org/), and FruitFly Splice Predictor (FF, https://www.fruitfly.org/seq_tools/splice.html) were employed to evaluate the effect of the mutation on pre-mRNA splicing. Subsequently, the impact on expression products was validated using MutationTaster (https://www.mutationtaster.org/), PolyPhen-2 (http://genetics.bwh.harvard.edu/pph2/), and VarCards (http://www.genemed.tech/varcards/).

### 2.4 Vector construction

Primer design: Two pairs of nested primers, 2-nested-F and 2-nested-R, 3-nested-F and 3-nested-R, were designed. Using normal human gDNA as the template, nested PCR was conducted to obtain two product templates, I2-1 and I2-2. The primer sequences are shown in Table 1.

1. The 584 bp wild-type N-terminal fragment was amplified from the *NFIX* CDS template using primers pCMV-3×Flag-Neo(EGFP)-*NFIX*-HindⅢ-F and *NFIX*-E2-I2-R, while the 792 bp wild-type C-terminal fragment was amplified using primers *NFIX*-I2-E3-F and pCMV-3×Flag-Neo(EGFP)-KpnI-R.
2. The 789 bp wt-1 fragment was amplified from the nested PCR product I2-1 and the wild-type N-terminal fragment using primers pCMV-3×Flag-Neo(EGFP)-*NFIX*-HindⅢ-F and *NFIX*-I2-R, while the 1041 bp wt-2 fragment was amplified from the nested PCR product I2-2 using primers *NFIX*-I2-F and pCMV-3×Flag-Neo(EGFP)-KpnI-R.
3. The HindⅢ-wt-KpnI fragment was amplified from wt-1 and wt-2 using primers pCMV-3×Flag-Neo(EGFP)-*NFIX*-HindⅢ-F and pCMV-3×Flag-Neo(EGFP)-KpnI-R. The wt fragment and pCMV-3×Flag-Neo(EGFP) vector were double-digested with HindⅢ and KpnI, ligated, and transformed to generate the pCMV-3×Flag-Neo(EGFP)-*NFIX*-wt vector, which was confirmed by sequencing.
4. Using wt as the template, the mutant’s first half segment mut-1 was amplified using pCMV-3×Flag-Neo (EGFP)-*NFIX*-HindⅢ-F and *NFIX*-mut-R as primers, and the mutant’s second half segment mut-2 was amplified using *NFIX*-mut-F and pCMV-3×flag-Neo (EGFP)-KpnI-R as primers.
5. Using mut-1 and mut-2 as templates, the wild-type HindⅢ-mut-KpnI fragment, the mut fragment with both HindⅢ and KpnI cuts, and the pCMV-3×Flag-Neo(EGFP) vector obtained by amplification with primers pCMV-3×Flag-Neo(EGFP)-*NFIX*-HindⅢ-F and pCMV-3×flag-Neo(EGFP)-KpnI-R were subjected to enzymatic digestion and then ligated. This resulted in the pCMV-3×Flag-Neo(EGFP)-*NFIX*-mut vector, which was verified by sequencing.

**TABLE 1:** The primers used in the vector construction.

Primer name	Primer sequence
2-Nested-F	CCG​CGA​GGA​CTT​CGT​GCT​GAC​CAT​CAC
2-Nested-R	cat​gcc​cct​tct​ctc​cac​cgg​act​cc
3-Nested-F	ctg​ggt​tgc​cag​gca​ctg​cct​ctg​ag
3-Nested-R	gct​tct​ccc​tcc​caa​gtc​ccg​ttt​cca​gc
pCMV-3×flag-Neo(EGFP)-*NFIX*-HindⅢ-F	tga​caa​gct​tAT​GTA​CTC​CCC​GTA​CTG​CCT
pCMV-3×flag-Neo(EGFP)-KpnI-R	gac​tgg​tac​cTC​AGA​AAG​TTG​CCG​TCC​CGG
*NFIX*-mut-F	AGG​ACG​AGG​AGC​GGG​GGT​GAA​GGA​CGA​GCT
*NFIX*-mut-R	AGC​TCG​TCC​TTC​ACC​CCC​GCT​CCT​CGT​CCT
*NFIX*-E2-I2-F	TTG​TCC​ACA​CTC​CGG​gta​ggt​cgt​tct​caa
*NFIX*-E2-I2-R	ttg​aga​acg​acc​tac​CCG​GAG​TGT​GGA​CAA
*NFIX*-I2-F	ttc​tat​tct​tcc​tcc​gag​aga​gca​atc​cag
*NFIX*-I2 -R	ctg​gat​tgc​tct​ctc​gga​gga​aga​ata​gaa
*NFIX*-I2-E3-F	ttg​tgt​ctc​ctg​cag​AAT​CCG​GAC​AAT​CAG
*NFIX*-I2-E3-R	CTG​ATT​GTC​CGG​ATT​ctg​cag​gag​aca​caa

### 2.5 Cell culture and transfection

HEK293T cells were obtained from the China Center for Type Culture Collection (CCTCC). The cells were cultured in DMEM medium supplemented with 10% fetal bovine serum (FBS). Cells were seeded into six-well plates 1 day prior to transfection. Transfection was performed when cell density reached 70%–90% confluence on the following day. Lipofectamine™ 3000 transfection reagent (Thermo Scientific, United States) was used for transfection. Incubated DNA-lipid complexes were added to the cells, gently mixed, and cultured in an incubator.

### 2.6 Quantitative real-time PCR (qPCR)

Cells were harvested 48 h post-transfection by trypsinization. Total RNA was extracted using TRIzol reagent, followed by phenol-chloroform purification. RNA concentration and quality were assessed using NanoDrop, and qualified samples were stored at −20 °C until use3. Residual genomic DNA was removed, and RNA was reverse-transcribed into cDNA using Hifair® Ⅱ 1st Strand cDNA Synthesis SuperMix for qPCR (gDNA digester plus) (Yeasen Biotechnology, Shanghai). cDNA products were immediately subjected to qPCR analysis. For quantitative real-time PCR (qPCR) analysis, β-actin served as the endogenous reference gene. Relative mRNA expression levels were determined using the comparative threshold cycle (2^−ΔΔCt^) method. The sequences of the qPCR primers were as follows: *NFIX*-qPCR-F: ggc​tct​aca​agt​cgc​ctc​ag, *NFIX*-qPCR-R: ccc​gga​agt​cac​aaa​aca​gt.

### 2.7 Western blot

HEK293T cells were seeded in six-well plates at a density of 1 × 10^6^ cells per well and cultured overnight at 37 °C with 5% CO_2_. Forty-eight hours post-transfection, cells were harvested and lysed with 100 μL RIPA lysis buffer (Institute of Biotechnology, China) supplemented with protease inhibitor (Roche Diagnostics, Switzerland) and phosphatase inhibitor (Shanghai Institute of Biotechnology, China). Total protein concentration was determined using the BCA assay kit (Tiangen Biotech, Beijing, China). Twenty micrograms of total protein per sample were separated on 12% SDS-PAGE gels and transferred to PVDF membranes (Bio-Rad, United States). Membranes were blocked with blocking buffer (Institute of Biotechnology, China) for 1 h at room temperature, followed by incubation with primary antibodies overnight at 4 °C: Anti-FLAG monoclonal antibody (Dianna BioTech, Wuhan, China; Cat# 2064; 1:1000 dilution); Anti-GAPDH Rabbit mAb (Cell Signaling Technology, United States; Cat# 2118S; 1:1000 dilution). Membranes were then incubated with species-matched HRP-conjugated secondary antibodies (Proteintech, United States; 1:2000 dilution) for 1 h at room temperature. Protein bands were visualized using SuperSignal West Pico PLUS Chemiluminescent Substrate (Thermo Fisher Scientific, United States) and quantified with ImageJ software.

### 2.8 Protein degradation pathway assay

Wild-type and mutant eukaryotic recombinant expression vectors were transiently transfected into 293T cells. After 48 h of transfection, cells were treated for 6 h with either: Autophagy-lysosome inhibitor: Chloroquine (CQ, 50 μM); Ubiquitin-proteasome inhibitor: MG-132 (50 μM). Both wild-type and mutant groups were subjected to inhibitor treatments. Cells were subsequently harvested, and degradation of truncated proteins was analyzed by Western blot.

### 2.9 Statistical analysis

Statistical analysis was performed using SPSS software (version 28.0). Quantitative data were expressed as mean ± standard deviation (Mean ± SD). For normally distributed data: Two-group comparisons: Independent samples t-test was applied. The significance level was set at α = 0.05 for all statistical tests.

## 3 Results

### 3.1 Whole exome sequencing results

**FIGURE 1:**
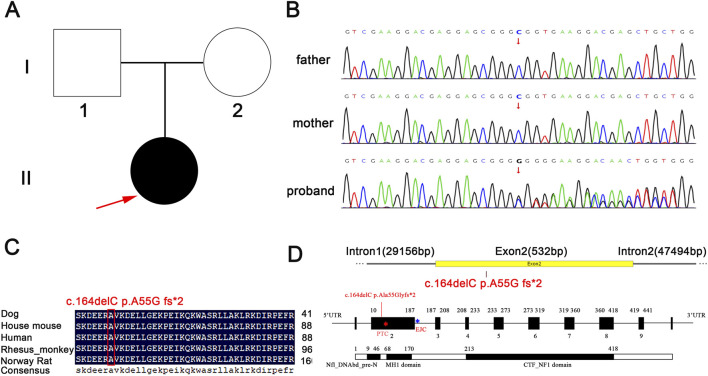
Family sequencing results and protein domain analysis. **(A)** Family pedigree analysis results. The red arrow indicates the proband. **(B)** Sequencing verification results of family members. **(C)** Conservation analysis of mutated amino acids. **(D)** The location of the mutated gene and the domain within the genome. Red * indicates the location of PTC, and blue * indicates the location of EJC.

The female proband was found to carry a heterozygous *NFIX* c.164delC p.Ala55Glyfs*2 mutation potentially associated with clinical phenotype. Targeted testing confirmed the absence of this *NFIX* mutation in both parents (mother and father), establishing its *de novo* origin (Figures 1A,B). According to the ACMG classification criteria, the *NFIX* mutation in the proband included evidence for PVS1 (the variant was a frameshift mutation and represented a loss-of-function variant) + PM2 (the variant was not identified in population databases such as ESP, 1000 Genomes, and ExAC), and was classified as a variant of likely pathogenic (LP). To definitively establish the pathogenicity of this *de novo* mutation, further *in vitro* experimental validation was necessary to generate novel pathogenic evidence.

### 3.2 Clinical phenotypic characteristics

The proband presented with intellectual disability, dysmorphic facial features, ocular abnormalities, scoliosis, and primary infertility at the Reproductive Center of Anhui Provincial Maternity and Child Health Hospital and underwent Preimplantation Genetic Diagnosis (PGD) for assisted reproduction. Intellectual disability, characteristic facial features, ocular abnormalities, and scoliosis are consistent with the clinical phenotype of Malan syndrome (Table 2). Peripheral blood karyotyping (400-band G-banding) revealed 46 chromosomes with normal structure and number. CT indicated scoliosis. Electrocardiogram (ECG) indicated sinus tachycardia and mild ST-segment abnormalities, while echocardiography showed no significant anomalies. Pelvic ultrasound demonstrated a normally sized uterus with homogeneous myometrium, midline endometrium, no adnexal masses or pelvic effusion, and no abnormal blood flow on color Doppler flow imaging (CDFI). Laboratory investigations revealed: Decreased serum 25-hydroxyvitamin D (25(OH)D: 15.82 ng/mL); Abnormal basal endocrine profile: Depressed luteinizing hormone (LH: 0.47 mIU/mL); Elevated estradiol (E_2_: 2139.13 pg/mL). Viral serology: Positive: HSV-I, rubella, CMV; Negative: HPV, HSV-II, toxoplasma. Urinalysis revealed elevated levels of erythrocytes (10/μL), leukocytes (15/μL), and epithelial cells (38/μL). Coagulation function was normal, and tumor marker CA125 was negative. Given primary infertility in both partners and maternal intellectual disability, embryo PGD was recommended to screen for heritable genetic mutations prior to *In Vitro* Fertilization (IVF).

**TABLE 2:** Clinical features of the proband and review of the other reported patients.

Characteristics	Present case	Malan et al.^a^			Yoneda et al.^b^	
	Pateint 1	Patient A	Patient B	Patient C	Patient 1	Patient 2
*NFIX* deletion/mutation	c.164delC	Del 19p13.3	Del 19p13.3	p.Q190X	p.L60P	p.R121P
Prenatal growth						
Sex	F	M	M	F	F	M
Birth weight (g)	-	4500(>95)	3110(10–50)	3600(50–90)	2816(10–50)	2938(10–50)
Birth height (cm)	-	53(95)	49(50)	52(95)	48.8(50)	51(50)
Present weight (g)	5400	-	-	-	-	-
Present height (cm)	163	-	-	-	-	-
Neurological						
Intellectual disability	+	+	-	-	+	+
Craniofacial features						
High forehead	+	+	+	+	+	+
Small mouth	+	+	-	+	nd	+
Connective tissue abnormalities						
Scoliosis	+	+	nd	+	+	+
Eye						
Strabismus	+	+	-	+	+	-

nd: no data available.

^a^ Reference 1;

^b^ Reference 17.

### 3.3 Mutation pathogenicity analysis

**FIGURE 2:**
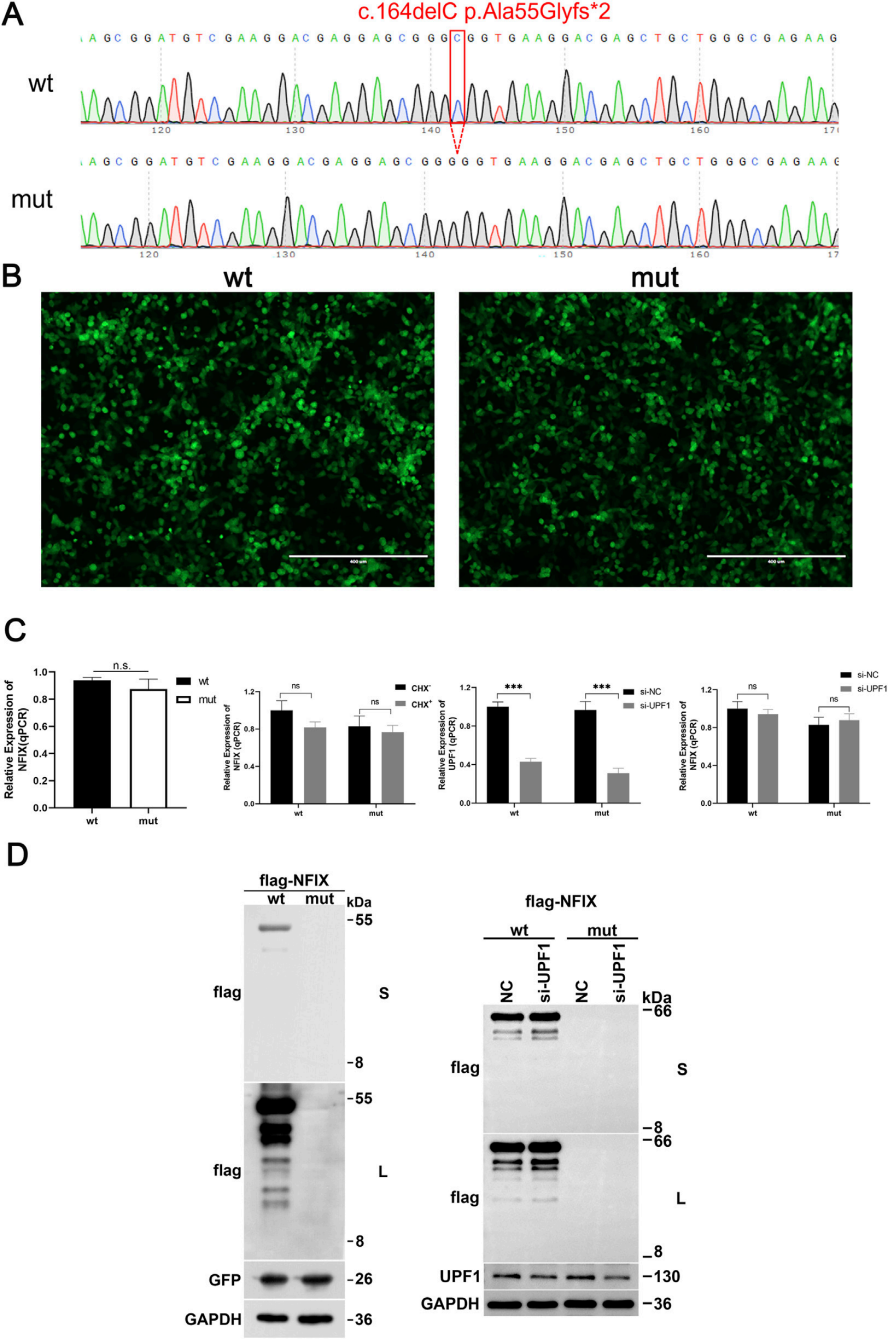
The influence of mutations on the expression levels of *NFIX* mRNA and protein. **(A)** Sequence results of expression vector construction. **(B)** Optimization of transfection efficiency assessment using GFP fluorescence. **(C)** The influence of mutations on the expression level of *NFIX* mRNA with CHX or si-UPF1 treatment. **(D)** The influence of mutations on the expression level of *NFIX* protein with si-UPF1. S stands for short exposure time, and L stands for long exposure time.

Protein sequence mutations in conserved regions often lead to loss of function or disease (Walker et al., 1999), while mutations in non-conserved regions drive adaptive evolution (Akand and Downard, 2018). Conservation reflects evolutionary selection pressure and serves as a key indicator for assessing the pathogenic risk of gene mutations and biological adaptability (Backwell and Marsh, 2022). The mutation *NIFX* c.164delC p.Ala55Glyfs*2 was analyzed using the PolyPhen-2 online tool. The 55th amino acid is highly conserved and is alanine in all five common species (Figure 1C). Based on NCBI and literature databases, the protein was determined to contain functional domains including NfI_DNAbd_pre-N, CTF_NFI, and MH1 (Figure 1D). With a total length of 441 amino acids, the mutation at position 55 was located outside all identified domains (Figure 2A). PolyPhen-2 analysis of the *NFIX* c.164delC p.Ala55Glyfs*2 mutation demonstrated that residue (alanine) was highly conserved across 14 species (Figure 2B), suggesting potential significant impacts on protein function. Pre-mRNA splicing impact was evaluated using multiple algorithms: RDDC^SC^: No alteration in splicing pattern compared to wild-type. SpliceAI: Minimal changed in confidence scores, indicating negligible splicing impact. FF: Unchanged splicing site scores, confirming no splicing disruption. MutationTaster predicted the variant as “disease-causing”, indicating altered amino acid sequence, frameshift, potential Nonsense-Mediated mRNA Decay (NMD), and compromised protein integrity (Table 3). Based on predictive outcomes from the aforementioned databases, the *NFIX* c.164delC p.Ala55Glyfs*2 variant was classified as a frameshift mutation predicted to trigger nonsense-mediated mRNA decay (NMD). This was expected to promote mRNA degradation and impair normal protein expression. These findings provided a basis for subsequent investigations into pathogenic mechanisms. Further analysis was conducted on the properties of the truncated protein. In the GPS-Uber prediction tool, the wild-type and mutant amino acid sequences (MYSPYCLTQDEFHPFIEALLPHVRAFSYTWFNLQARKRKYFKKHEKRMSKDEERG) were input, and the possible ubiquitination sites were identified as 57, 64, 111, and 152, with Uber scores of 0.3927, 0.3931, 0.3408, and 0.5726, respectively (Table 4). After the mutation, the ubiquitination sites were absent.

**TABLE 3:** Bioinformatics predictions of the proband and review of the other reported patients.

Bioinformatics predictions	Present case	Malan et al. 2010			Yoneda et al. 2012	
	Pateint 1	Patient A	Patient B	Patient C	Patient 1	Patient 2
*NFIX* deletion/mutation	c.164delC	Del 19p13.3	Del 19p13.3	p.Q190X	p.L60P	p.R121P
Splicing function						
SpliceAI	Not affected	NA	NA	Not affected	Not affected	Not affected
FF	Not affected	NA	NA	Not affected	Not affected	Not affected
RDDC^sc^	Not affected	NA	NA	Not affected	Not affected	Deleting 184 bp, frameshift mutation
Known variant						
gnomAD	Not included	NA	NA	Not included	Not included	Not included
HGMD	Not included			disease mutation (CM105133)	disease mutation (CM124376)	disease mutation (CM124377)
Clinvar	Not included			Pathogenic (ID 36956)	Pathogenic (ID 36966)	Pathogenic (ID 36967)
Effect of expression products						
Mutation taster	Deleterious, NMD	NA	NA	Deleterious, NMD	Deleterious	Deleterious

NA: Not applicable.

**TABLE 4:** Result of ubiquitin-E3 enzymes relationship predicit

Wt position	Peptide	Score	Mutant state
57	RMSKDEERAVKDELLGEKPEI	0.3927	Lost
64	RAVKDELLGEKPEIKQKWASR	0.3931	Lost
111	PCCVLSNPDQKGKIRRIDCLR	0.3408	Lost
152	LESTDGERLYKSPQCSNPGLC	0.5726	Lost

### 3.4 Impact of the mutation on mRNA and protein expression levels

Quantitative real-time PCR (qPCR) analysis demonstrated that in the pCMV-3×Flag-Neo(EGFP)-*NFIX* plasmid series (Figure 2A). The GFP fluorescence intensity suggested no statistically significant difference in transfection efficiency between the wt and mut groups (Figure 2B). The mRNA expression level of the mutant type (c.164delC p.Ala55Glyfs*2) showed no significant difference compared to the wild type (Figure 2C, *P* > 0.05). CHX or si-UPF1 treatment also showed same results (Figure 2C, *P* > 0.05). Western blot results showed that the theoretical size of the wild-type protein was 66 kDa and the theoretical size of the mutant protein was 8 kDa in the pCMV-3×Flag-Neo (EGFP)-*NFIX* series vectors. The wild-type protein was of the expected size and had a single band, and the mutant protein did not detect a band at the corresponding position (Figure 2D). Western blot analysis failed to detect the mutant protein at the expected molecular weight, indicating that the mutant protein may be degraded through other pathways. Since there was no suitable positive marker for the CHX treatment, only the response effect of si-UPF1 was observed. The results showed that the si-UPF1 treatment had no significant effect on the expression level of the *NFIX* protein. Further mechanistic studies were warranted to delineate specific degradation pathways.

### 3.5 Mutation protein degradation pathway

**FIGURE 3:**
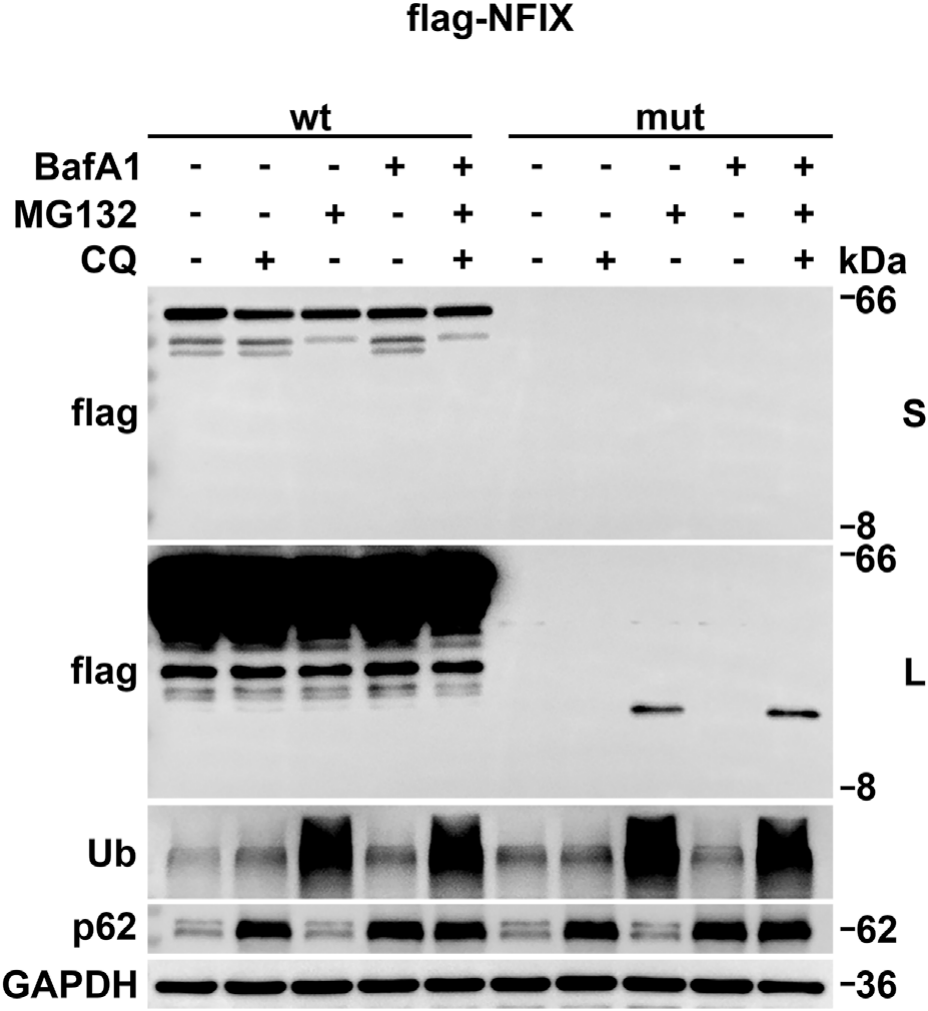
The degradation pathway of the mutant protein by Western blot with CQ, MG132, BafA1 treatment. Ubiquitinated protein (Ub) accumulation was added as a positive control to confirm proteasome inhibition. p62 protein degradation was monitored to validate autophagy-lysosomal pathway inhibition. S stands for short exposure time, and L stands for long exposure time.

Western blot results revealed that the wild-type protein size matched the expected value, whereas the mutant protein size was larger than predicted but exhibited a single band. *NFIX* expression in the mutant group was significantly downregulated compared to the wild-type group, with no band detected in the mut (NC) group. The autophagic inhibitor Chloroquine (CQ) or lysosomal inhibitor BafA1 showed no significant effect on *NFIX* expression in either wild-type or mutant groups. In contrast, the ubiquitin-proteasome pathway inhibitor MG-132 had no significant impact on wild-type expression but significantly reversed the downregulation of *NFIX* protein expression in the mutant group (Figure 3). The combined application of several inhibitors (CQ, BafA1, MG-132) can inhibit various pathways of protein degradation, excluding the influence of translation efficiency. These inhibitor-based rescue experiments confirmed that *NFIX* protein degradation occurs predominantly through the ubiquitin-proteasome pathway. The degradation of the truncated protein leads to reduced *NFIX* expression, which may contribute to disease pathogenesis.

## 4 Discussion

Malan syndrome is an autosomal dominant disorder characterized by: Postnatal overgrowth (height/weight >97th percentile) and dysmorphic craniofacial features (Kuroda et al., 2017); Neurodevelopmental impairments, including mild-to-moderate intellectual disability, delayed speech development, and autism spectrum traits (Huynh et al., 2024); Connective tissue abnormalities, notably scoliosis and joint hyperlaxity (Priolo et al., 2018). Definitive diagnosis of Malan syndrome requires meeting both criteria: (1) ≥2 major clinical features (postnatal overgrowth with height/weight >97th percentile, neurodevelopmental disorders, characteristic craniofacial dysmorphism), and (2) identification of a pathogenic *NFIX* variant classified as Pathogenic/Likely Pathogenic under ACMG guidelines (Macchiaiolo et al., 2022). This study reported a Malan syndrome pedigree caused by an *NFIX* mutation. The proband in this pedigree underwent preimplantation genetic diagnosis during IVF treatment due to intellectual disability, dysmorphic features, ocular abnormalities, scoliosis, and primary infertility. Although the patient exhibited the cardinal features of Malan syndrome-intellectual disability, characteristic facial dysmorphism, and thoracolumbar scoliosis-his height (163 cm) and weight (54 kg) deviated from the expected overgrowth phenotype. Primary infertility may be caused by abnormal hormone metabolism during pregnancy. To achieve a definitive diagnosis, whole-exome sequencing (WES) was performed on the proband’s specimen, which identified the phenotype-associated *NFIX* c.164delC (p.Ala55Glyfs*2) mutation. The proband’s intellectual disability aligns with Malan syndrome phenotypes, while her primary infertility may relate to abnormal prenatal hormone metabolism. Genetic validation in the pedigree confirmed that: Neither parent of the female proband carried the mutation, indicating a *de novo* origin. The variant was classified as “likely pathogenic”. This mutation was absent from gnomAD and HGMD databases, necessitating clarification of its genotype-phenotype correlation to inform embryo screening for *in vitro* fertilization.

The *NFIX* gene encodes a member of the nuclear factor I (NFI) transcription factor family (including NFIA, NFIB, NFIC) (Fraser et al., 2020), which regulates target gene expression by binding DNA palindromic sequences and participates in cell proliferation, differentiation, and organ development (Matuzelski et al., 2017). *NFIX* mutations are primarily associated with two autosomal dominant disorders: Marshall-Smith syndrome and Malan syndrome (Mulder et al., 2020). Pathogenic variants in Malan syndrome predominantly consist of protein-truncating mutations (nonsense/frameshift) (Uzman et al., 2023), alongside rare missense (Klaassens et al., 2015) and splice-site mutations (Yoneda et al., 2012). The *NFIX* c.164delC (p.Ala55Glyfs*2) variant identified in our study represented a loss-of-function truncation, which-consistent with prior reports (Huynh et al., 2024; Priolo et al., 2018)-was predicted to cause haploinsufficiency-driven pathogenesis, explaining the neurodevelopmental deficits. ClinVar currently annotates NM_001365902.3(*NFIX*).163del (p.Ala55fs) as causing Marshall-Smith syndrome, while our functional and clinical data demonstrate that NM_001365902.3(*NFIX*) 164delC (p.Ala55Glyfs*2) shows stronger correlation with Malan syndrome. According to the OMIM database, *NFIX* gene mutations can cause two clinically overlapping syndromes: Malan syndrome and Marshall-Smith syndrome. The main clinical features of Malan syndrome include: childhood overgrowth; characteristic long face; joint laxity; ventriculomegaly. In contrast, Marshall-Smith syndrome is primarily characterized by: accelerated bone maturation; respiratory distress; distinctive facial features.

In order to clarify the pathogenicity of this *de novo* mutation, the effect of the mutation on the structural domain of the protein was first analyzed, and the results showed that the *NFIX* protein has the functional structural domains NfI_DNAbd_pre-N, CTF_NFI, and MH1, which has a length of 441aa, and the mutation was on Exon2, which was located outside all identified domains (Yoneda et al., 2012). This structural domain was consistent with the finding that the mutations causing Malan syndrome are concentrated in exon 2 and exon 3, while the mutations causing the more severe Marshall-Smith syndrome are distributed from exon 6 to exon 10 (Martinez et al., 2015). Further analysis of the effect of the mutation on splicing function by bioinformatics showed that databases such as SpliceAI, FF, and RDDC^SC^ predicted that the mutation did not affect splicing, and MutationTaster predicted that the mutation resulted in altered amino acids, altered reading frames, and possible NMD, affecting the protein characteristics. Based on this, it was hypothesized that the mutation may cause mRNA degradation through other NMD pathways, leading to Malan syndrome.

Next, the effect of the mutation on *NFIX* mRNA expression levels was examined by qPCR, and the results showed that the mutation did not lead to a decrease in mRNA levels compared with the wild type. However, Western blot results showed that the mutation significantly decreased *NFIX* protein levels compared to the wild type, and no truncated protein was detected. Neither CHX nor si-UPF1 treatment could restore the protein expression level of *NFIX*. The absence of mRNA reduction coupled with markedly diminished protein levels implies that the truncated protein escapes nonsense-mediated decay (NMD) and was instead targeted by alternative degradation mechanisms, such as the ubiquitin-proteasome system or autophagy. These two major proteolytic systems-the autophagy-lysosome system and the ubiquitin-proteasome system-constitute the core machinery for selective clearance of dysfunctional proteins. Through chaperone-mediated targeting, they degrade misfolded/damaged proteins (Sandri, 2013), prevent toxic oligomer formation (Mauthe et al., 2018), and maintain proteostasis via integration with stress-response pathways (Zheng et al., 2024). In this study, the truncated protein generated after the *NFIX* mutation was predicted by GPS-Uber to lack degradation signal motifs or lysine residues that mediate ubiquitination. The mutant protein truncated by the premature termination codon (PTC), loses its C-terminal functional domain, exposing originally buried hydrophobic residues or misfolded regions. These aberrant structures are recognized by molecular chaperones (Hsp70) and subsequently delivered to E3 ubiquitin ligases, thereby initiating ubiquitination tagging.

In order to clarify the pathway through which truncated proteins are degraded, the autophagy-specific inhibitor CQ (Mauthe et al., 2018) lysosome-specific inhibitor BafA1 (Zheng et al., 2024), and the ubiquitin-proteasome pathway inhibitor MG132 (Chitra et al., 2012) were used to inhibit these degradation pathways. The results showed that the inhibitor of autophagic pathway CQ or lysosome-specific inhibitor BafA1 had no significant effect on the expression of *NFIX* in the wt group and the mut group, and there were still no bands in the mut (CQ or BafA1) group. While the ubiquitin-proteasome pathway inhibitor MG-132 had no significant effect on *NFIX* expression in the wt group, but significantly reversed the downregulation of *NFIX* protein expression in the mut group. Inhibit almost all protein degradation pathways (CQ + BafA1+MG132 treatment) to eliminate the influence of translation efficiency. It is suggested that the wild-type *NFIX* protein was not degraded, while the *NFIX* truncated protein was may degraded through the ubiquitin-proteasome pathway. Other potential mechanisms such as protein aggregation or co-translational degradation, which could be further investigated using methods like native electrophoresis or ribosome profiling in future studies.

Degradation of the truncated protein via the ubiquitin-proteasome results in a single underdose, which leads to the development of Malan syndrome. This is consistent with Malan’s report of a mutation located in Exon2 to Exon4 that leads to the Malan syndrome phenotype by triggering the NMD pathway (Malan et al., 2010). Although the mutation did not trigger the NMD pathway, the truncated protein was degraded via the ubiquitin-proteasome pathway, which also caused a single underdose effect. The current study suggests that *NFIX* mutations triggering or evading NMD can lead to two different clinical phenotypes, Malan syndrome or Ma Shi syndrome, with Malan syndrome having overgrowth as the main phenotype (Makker et al., 2024) caused by a major *NFIX* single-dose insufficiency. In contrast, the presence of normal and mutant alleles in patients with Ma Shi syndrome supports the escape of mutant RNAs from NMD surveillance (Mendell and Dietz, 2001; Holbrook et al., 2004; Chang et al., 2007), and *NFIX* mutations lead to the production of mutant proteins in MSS, which can have a dominant-negative effect on the wild-type allele (Kooblall et al., 2023), ultimately leading to a more severe phenotype.

The ClinVar entry for NM_001365902.3(*NFIX*).163del (p.Ala55fs) only describes clinical characteristics without further molecular mechanism validation. Our study not only explains the relationship between *NFIX* mutation (NM_001365902.3(*NFIX*).164delC (p.Ala55Glyfs*2)) and Malan syndrome from the perspective of genotype-phenotype correlation (the proband showed no Marshall-Smith features like accelerated bone maturation or respiratory distress, but exhibited Malan syndrome characteristics including facial dysmorphism, intellectual disability, and scoliosis), but also elucidates the molecular mechanism through proteasomal degradation leading to *NFIX* haploinsufficiency. According to the ACMG guidelines, PS3 evidence (functional studies confirm the variant severely impairs gene/protein function) and PM1 evidence (haploinsufficiency was a well-established disease mechanism) were added, allowing the pathogenicity classification to be upgraded to “pathogenic”. Given the autosomal dominant (AD) inheritance pattern of *NFIX* mutations, there is a higher probability of offspring inheriting this pathogenic variant. It is recommended to design specific probes targeting this mutation to accurately screen healthy embryos and utilize Third-Generation *In Vitro* Fertilization (IVF) technology for selecting unaffected offspring.

In summary, this study reported a Malan syndrome pedigree caused by a frameshift mutation in the *NFIX* gene. The proband exhibited intellectual disability but no significant overgrowth phenotype. This frameshift mutation did not significantly affect mRNA expression levels but led to protein degradation via the ubiquitin-proteasome pathway, resulting in haploinsufficiency and ultimately causing Malan syndrome.

## Data Availability

The original contributions presented in the study are included in the article/supplementary material, further inquiries can be directed to the corresponding author.
